# The experiences and perceptions of female breast cancer patients regarding weight management during and after treatment for oestrogen-receptor positive disease: a qualitative study

**DOI:** 10.1186/s12885-022-10238-7

**Published:** 2022-11-18

**Authors:** Saxton JM, Pickering K, Wane S, Crank H, Anderson AS, Cain H, Cohen J, Copeland RJ, Gray J, Hargreaves J, McNally RJQ, Wilson C

**Affiliations:** 1 School of Sport, Exercise & Rehabilitation Sciences, Faculty of Health Sciences, University of Hull, Cottingham Road, Hull, HU6 7RX UK; 2 Advanced Wellbeing Research Centre, Sheffield Hallam University, Olympic Legacy Park 2 Old Hall Rd, Sheffield, S9 3TU UK; 3 Department of Sport, Exercise & Rehabilitation, Northumbria University, City Campus, Newcastle-Upon-Tyne, NE1 8ST UK; 4 Academy of Sport and Physical Activity, Sheffield Hallam University, Sheffield, S10 2BP UK; 5 Division of Population Health & Genomics, Centre for Research Into Cancer Prevention and Screening, Ninewells Hospital and Medical School, University of Dundee, Dundee, DD1 9SY UK; 6 Newcastle Upon Tyne Hospitals NHS Foundation Trust, Queen Victoria Road, Newcastle Upon Tyne, NE1 4LP UK; 7 York Medical School, Hull Heath Trials Unit, University of Hull, Cottingham Road, Hull, HU6 7RX UK; 8 Department of Nursing, Midwifery & Health, Northumbria University, Coach Lane Campus, Newcastle Upon Tyne, NE7 7XA UK; 9 Carnegie School of Sport, Leeds Beckett University, 9 Fairfax Hall, Headingley Campus, Leeds, LS6 3QS UK; 10 Population Health Sciences Institute, Newcastle University GB, Newcastle Upon Tyne, NE1 7RU UK; 11 Department of Oncology and Metabolism, The Medical School, University of Sheffield, Beech Hill Road, Sheffield, S10 2RX UK

**Keywords:** Breast Cancer, Hormone-positive, Weight management, Barriers and facilitators

## Abstract

**Background:**

Weight gain is commonly observed during and after breast cancer treatment and is associated with poorer survival outcomes, notably in women with oestrogen-receptor positive disease. The aim of this qualitative study was to investigate the experiences and perceptions of oestrogen-receptor positive (ER +) female breast cancer patients (BCPs) regarding weight management behaviours during and after treatment. Secondly, to gain insight into the experiences of healthcare professionals (HCPs) regarding the provision of weight management advice to patients undergoing treatment.

**Methods:**

Four focus groups involving 16 BCPs having a median (range) age of 51 (35–70 y) and three focus groups involving 21 HCPs aged 46 (29–62) were held at a university campus, local cancer support centre or clinical site. Data were analysed using Framework analysis.

**Results:**

Four overarching themes (and 10 subthemes) were identified: (1) Treatment; (2) Support for lifestyle behaviour change; (3) Information availability for BCPs; (4) Knowledge of current evidence amongst HCPs. The physical and psychological consequences of treatment influenced motivation for weight management amongst BCPs. Social support for health promoting behaviours was viewed as important but was conflicting, requiring context-specific considerations. BCPs said they would have welcomed access to credible information (guided by HCPs) about the potential detrimental health effects of excess body weight and weight gain, together with advice on weight management via healthy eating and physical activity. HCPs felt that they had insufficient knowledge of public health dietary and physical activity recommendations or evidence-based interventions to confidently offer such advice. HCPs expressed concern that raising weight management issues would exacerbate distress or invoke feelings of guilt amongst BCPs, and cited time pressures on patient consultations as additional barriers to providing weight management support.

**Conclusion:**

The study yielded novel insights into factors influencing weight management behaviours amongst overweight ER + BCPs. The results suggest that evidence-based information and support, which addresses key physical and psychological challenges to physical activity and dietary behaviours, offers the best route to sustainable weight management in this population.

**Supplementary Information:**

The online version contains supplementary material available at 10.1186/s12885-022-10238-7.

## Background

Weight gain is commonly observed during and after breast cancer treatment due to chemotherapy and endocrine therapies, induced menopause, changes in metabolism and food intake and decreased physical activity [1, 2]. Systematic review evidence shows that women who are overweight and obese at diagnosis, and those who gain weight, have poorer breast cancer survival outcomes than women of a healthy weight, irrespective of menopausal status [3]. Excess body weight after breast cancer also increases the risk of type 2 diabetes mellitus and cardiovascular disease [4, 5].

The adverse impact of excess body weight on survival outcomes is clearly shown for women with oestrogen-receptor positive (ER +) breast cancer [2, 6], which accounts for 70% of all breast cancer cases [7]. Higher body fat increases the risk of ER + recurrence because of increased aromatase activity and circulating levels of oestrogens and androgens [8]. This is compounded by other risk factors, including abnormal insulin and adipokine metabolism, impaired anti-tumour immunity and chronic low-grade systemic inflammation [2, 9]. Although associations between intentional weight loss and survival outcomes after primary breast cancer treatment are not yet well established [10, 11], the significant body of observational evidence pointing to the adverse impact of weight gain provides a strong rationale for the development of accessible and adoptable weight management interventions.

Previous qualitative studies have identified a range of important barriers to adopting and adhering to health promoting behaviours amongst breast cancer patients (BCPs). These include treatment-related physical symptoms which impede physical functioning (e.g., lymphoedema which restricts upper-limb range of motion), fatigue, pain, lack of confidence, body image concerns, fears about health behaviour change due to feelings of vulnerability, co-morbidities and conflicting priorities (e.g., work commitments, family caring duties, etc.) and low motivation [12–15]. In contrast, important facilitators have been identified as desire to lose weight [14], access to supervised exercise and dietary education, feeling a sense of control, peer support and having an opportunity to regain a sense of normality [16]. Women commonly experience deficits in the availability of clear and simple information on lifestyle, citing insufficient support from healthcare professionals (HCPs) [15]. Group-based interventions can provide an opportunity for peer-to-peer support as a means of addressing barriers to health-promoting behaviours and building the skills and confidence needed for dietary and physical activity behaviour change. Successful group-based weight-loss interventions in breast cancer patients have used a variety of delivery formats, including face-to-face workshops for 8–15 women alongside remote support methods such as telephone, emails, text-messaging and printed mail-outs [17–21].

In the UK, support for health behaviour change after primary treatment for breast cancer is limited to that provided by prominent cancer charities. For example, Breast Cancer Now offer an on-line course and book/printed materials (“Moving Forward”) to help women adjust to life after breast cancer treatment. This takes place over half a day for three or four weeks and aims to provide information, support and professional guidance on how to cope with and adjust to life after breast cancer treatment [22]. Macmillan Cancer Support offer the Recovery Package after completion of primary treatment comprising a Holistic Needs Assessment, treatment and cancer care reviews with a HCP and an education/support event such as a Health and Wellbeing Clinic [23]. There remains a gap however, in longer-term provision of tailored (bespoke) lifestyle support, specifically designed to address the barriers to effective weight loss that many women experience after primary treatment for breast cancer. This means that offering a route to accessible and adoptable weight management support would address an important unmet need for women and their treating clinicians at what is frequently an opportune ‘teachable moment’ for patients [24].

Understanding and addressing the challenges women face adopting and adhering to healthy lifestyle behaviours during and after breast cancer treatment from the perspective of both BCPs and HCPs is an important first step in designing effective and sustainable weight loss interventions for this population. Current guidelines for cancer survivors recommend achieving/maintaining a healthy body weight, engaging in regular physical activity and eating a healthy dietary pattern that is high in vegetables, fruits and whole grains and low in calorific foods and beverages, processed meats and alcohol [25–27]. However, recent survey evidence showed that while a high proportion of breast cancer patients reported at least one positive nutrition or physical activity behaviour after diagnosis or treatment, several treatment-related barriers (fatigue, stress, changes in appetite/taste disturbances, pain and discomfort) impeded the adoption of these health behaviours [28]. Furthermore, a recently published American College of Clinical Oncology Guideline paper emphasised the need for more research into diet and weight management strategies for people undergoing cancer treatments [29]. Thus, the aim of this qualitative study was to investigate the experiences and perceptions of overweight ER + BCPs regarding weight management (healthy dietary behaviours and physical activity) during and after primary treatment and ongoing hormone therapy. Secondly, to gain insight into the experiences of HCPs regarding the provision of weight management advice to BCPs undergoing treatment for ER + disease.

## Methods

### Participant recruitment

A purposive sample of female breast cancer patients attending routine follow-up clinical visits were invited to participate in the study by members of their clinical team or a research nurse. Women were eligible to participate in the study if they were over 18 years of age and were more than eight weeks since completion of chemotherapy (providing time to reflect on their experience of adjuvant treatment) and less than 36 months since completion of primary treatment for ER + breast cancer (for accurate recall of experiences), with a BMI ≥ 25 kg/m^2^. Women being prescribed hormone therapies were eligible. HCPs from five UK National Health Service (NHS) Trusts were recruited by the lead investigators. Emails containing details of the study were sent to HCPs involved in the care of breast cancer patients and they were invited to respond to the research team for further information.

### Procedures and data collection

Our underlying philosophy was constructivist [30], recognising the individual nature of experience and the impact of BCPs’ wider life experiences on their perspectives of weight management behaviours. Four focus groups involving 16 BCPs and three focus groups involving 21 HCPs were held at a university campus, a local cancer support centre or at a clinical site between December 2018 and January 2019. Each session lasted between 70 and 89 min (mean = 81 min). Initially, two HCP focus groups took place, however, due to emerging qualitative data highlighting the importance of appropriate and safe lymphoedema management, an additional focus group was arranged with lymphoedema practitioners. The focus groups were led by an experienced female qualitative researcher (either SW, a physiotherapist or KP, a physical activity public health expert, both were qualified in exercise prescription for cancer patients), with assistance from at least one other member of the research team (HC, SW, KP or JS), and all sessions were audio-recorded. Topic guides were developed on the basis of study objectives, previous literature and knowledge of important issues for BCPs amongst members of the research team gleaned from previous research and clinical practice. Topic guides explored barriers and facilitators to weight management (healthy eating and physical activity) from the perspectives of BCPs and HCPs during and after ER + breast cancer treatment (Table 1). Participant characteristics are presented in Table 2. All participants provided written, informed consent prior to data collection and the study was approved by the Northwest Preston NHS Research Ethics Committee (18/NW/0400). The research was conducted and reported in accordance with qualitative research reporting guidelines [31].

**Table 1:** Focus group topic guide

Topic guide for patients	Topic guide for healthcare professionals
**Thoughts and experiences of weight management before and after breast cancer diagnosis:** What, if any, has been your experience of weight loss before/after to your cancer diagnosis? What diets did you try? Did they work for you? If so why, and if not, why not? What have been the main challenges of managing weight since your treatment? Are you experiencing any symptoms related to your treatment? If so, what are these? Do these symptoms have an effect on your motivation to lose weight, or have they had an effect on any attempts you have made to lose weight?	**Perceptions of weight gain amongst patients during treatment:** What proportion of your patients with hormone-positive breast cancer are overweight/obese at point of diagnosis? What happens to women's weight status during treatment and post-treatment? In your opinion do hormonal therapies or medications have any impact on a patient’s weight/attempts to lose weight/maintain weight loss? In your opinion do chemotherapy have any impact on a patient’s weight/attempts to lose weight/maintain weight loss?
**Understanding dietary behaviours:** What do you think about diets? Have you tried dieting since your treatment? Did your clinical team encourage/endorse diet modification for helping with weight management following treatment? Do those in your social circle (family and friends) support you when trying to manage your weight? How can we help women who are living with and beyond cancer to make changes to their diet that could help with weight management / loss?	**Current practice:** Do you have conversations with patients around healthy eating, weight management and exercise? If so, who initiates these conversations – would you always initiate such conversations, or does it happen in response to patient questions? Is any advice given “general” or do you tailor the information that you give to patients? Do you recommend weight management or exercise to manage certain symptoms associated with cancer or its treatment? Can you refer patients to community-based weight management or exercise programmes?
**Understanding exercise or physical activity behaviours:** Do you currently engage in physical activity or exercise? Did your clinical team encourage you to be physically active / endorse or advise exercise or physical activity for helping with rehabilitation or recovery? Did/Do you have apprehensions about being physically active or exercising? Do those in your social circle (family and friends) support you when trying to be active? What are the important questions you need answering if you are thinking about becoming physically active or exercising?	**Weight management advice/information requested by patients:** Are you asked by patients for specific advice on being physically active or exercising pre-treatment/ during or post-treatment? What type of information do patients ask for? Do patients ask about the risk of being overweight or physically inactive relative to disease recurrence? Do any Public Health Guidelines or cancer specific lifestyle guidelines ever feature in conversations/consultations?
**Technology and weight management:** Do you use technology in your daily life, e.g. websites, smartphone apps etc.? Have you ever used any technologies to help you manage your weight, follow a diet or to motivate you to be more physical activity such as on-line programmes, smartphone apps, wearable devices?	**Perceived benefits of weight management advice for patients:** Have you seen any benefits in patients from becoming or already being physically active/being an ideal body weight/BMI during or after treatment? If so, what are those benefits? Why do you think some patients are more successful than others in managing their weight and activity levels?
	**How the provision of weight management advice could be improved:** How prominent is lifestyle modification such as healthy eating and exercise a feature of clinical care/survivorship care? Do you feel confident about giving advice on healthy eating and exercise? What would help you increase your confidence to give advice? What would support your own practice? What limits your ability to deliver advice about weight management and exercise? What are the barriers to initiating or holding a conversation on these topics? What is needed to enable more health professionals to have more frequent (routine) conversations about healthy eating and exercise with their patients?

**Table 2:**
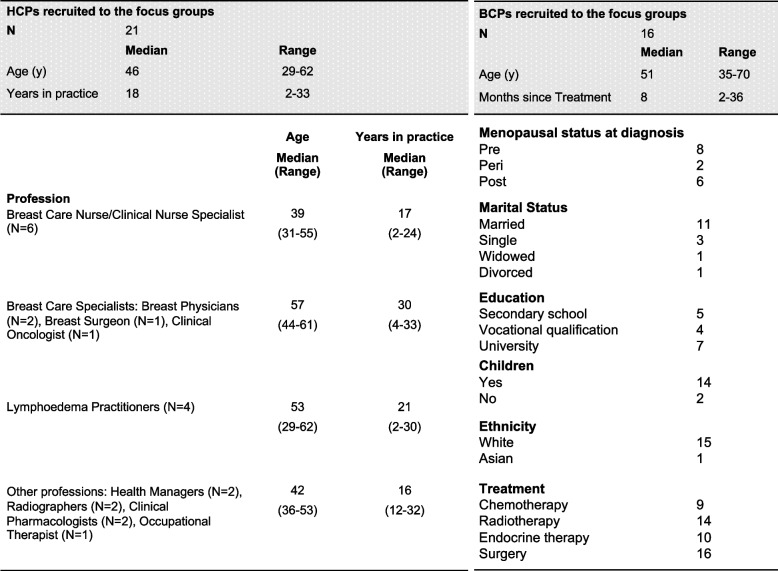
Participant characteristics

### Data analysis

Audio-recordings were transcribed anonymously, verbatim. Two researchers independently analysed the data using framework analysis [32], comprising: (i) familiarisation with the data; (ii) identification of a thematic framework (informed by a combination of a priori topics and emergent issues); (iii) indexing (i.e. applying framework codes to the data); (iv) charting (i.e. summarising indexed data); and (v) mapping and interpreting the data (assessing meaning, differences and similarities etc.) [33]. Transcripts were initially analysed and charted in relation to the a priori topics that guided focus group discussions, with the BCP and HCP focus groups coded into this framework separately. Commonalities and differences between the views and experiences of BCPs and HCPs were used to create overarching themes and subthemes, before the data from the two participant groups were coded in relation to one another. NVivo (version 11; QSR International, Melbourne, Australia) was used to organise the data. At each stage of the process the guiding framework was modified in relation to the emergent content within the data. The researchers liaised at several points to discuss data saturation, which was confirmed during thematic analysis when no new codes, categories or themes emerged from the data. The coding framework represented all relevant data, and there was a high level of agreement between analysers. The emergent themes were discussed and the final themes refined. This approach to data analysis was somewhat deductive, framing the analysis within the a priori topic guide, yet the data were borne out of original transcripts from interviews and focus groups [34].

## Results

Four overarching themes (and 10 subthemes) were identified: (1) Treatment; (2) Support for lifestyle behaviour change; (3) Information availability for BCPs; (4) Knowledge of current evidence amongst HCPs. Example quotes from BCPs and HCPs are used to illustrate each theme (and subtheme), with the latter also including a gender identifier in parentheses (F or M). Lymphoedema practitioners (all female) are denoted by the letter L. A more complete list of quotes to illustrate each theme (and subtheme) is presented in the Supplementary Table.

### Theme 1: treatment

The physical and psychological impacts of treatment emerged as an important theme negatively influencing motivation for weight management behaviours amongst BCPs. BCPs and HCPs considered the medications prescribed during treatment, the psychological impact of a cancer diagnosis and altered dietary patterns due to taste disturbances, altered food choices, comfort eating and the perceived convenience of, or craving for, less healthy foods (e.g., sugary foods) to be important factors impeding motivation for healthy lifestyle behaviours.

#### Side-effects of treatment

Several side-effects of breast cancer treatment have the potential to influence weight gain. BCPs talked about how their side-effects impacted their activity levels and diet.

BCP8: [After surgery] I couldn’t go swimming, and I can’t do yoga because I couldn’t move my arms up... I spent a week sitting on the sofa because I’m carrying two drains around with me. There’s nothing you can do. So, your weight starts to move and how quickly it starts to move is just, was quite depressing, and doesn’t help your mood, which doesn’t help what you eat… and you think well why should I bother?

BCP15: In terms of my diet, it was the chemotherapy that just messes up your digestive system completely, so I was just eating whatever I fancied, your taste has gone horrible and your digestion’s gone rubbish and you just eat what you can when you can... the nurse is joking saying it’s the opposite of Weight Watchers here, we want you to put on weight, and everything.

HCPs said they make patients aware of the side-effects of hormone therapies during patient consultations and these messages are reinforced via printed literature. Some HCPs provided very general advice on how to counteract common side-effects, such as loss of bone mineral density.

*HCP(M)16: And I think for those of us who are initiating certainly aromatase inhibitors, we will of necessity warn them of the risk of osteoporosis... and as part of that would be to say the most useful thing you can do to avoid it is to be as active as possible. So, we would say that.*

#### Pre-treatment expectations

BCPs were under the impression that they would lose weight during their treatment and were surprised that they gained weight (all reported gaining weight during treatment).

BCP4: I had a misconception that I was going to lose weight on chemotherapy, and it was exactly the opposite.

L1: One thing I do hear quite often from ladies when they’re going through their cancer journey, the one thing they’ll say is I thought I’d lose loads of weight – because they had this idea of someone with cancer being emaciated. And actually, that’s not the case with most of them...

#### Prioritisation of treatment

The adoption of healthy lifestyle behaviours for weight management was regarded as low priority during treatment because of the physical and emotional strain of the breast cancer diagnosis and disruption to normal routines imposed by the treatment.

BCP2: I put on two stone during treatment… part of that was the steroids, but part of that was the oh shit I’m going to die so I might as well eat cake, because why wouldn’t you do that?

HCP(F)1: Their normal pattern goes out of the window, and they’re just trying to get through the treatments, and diet isn’t their priority, nor exercise.

### Theme 2: support for lifestyle behaviour change

Support for lifestyle behaviour change from HCPs along the breast cancer treatment pathway was perceived to be minimal amongst BCPs. For some BCPs, motivation to engage with weight management behaviours (particularly physical activity) was strongly influenced by the support of significant others, including their peer-group (other BCPs). However, for some BCPs, friends and family tended to be over-protective, and others wanted to ‘move on’ from their cancer experience which made them reluctant to seek support from their peer-group.

#### Support within the clinical pathway

There was a perceived lack of support from HCPs, and contrary to promoting healthy lifestyle behaviours, there were some instances of HCPs encouraging BCPs to do the opposite.

BCP8: Indulge is what the chemo nurses said. If you want that piece of cake, you have that piece of cake. Thanks ladies.

BCP15: [HCPs] just told me not to worry about it… and it was good to put on weight during chemo rather than losing weight, that was what they said…

Other HCPs expressed their reservations about providing support for lifestyle behaviour change during treatment when women are vulnerable and distressed due to their recent breast cancer diagnosis. They expressed concerns about raising weight management issues during treatment because they did not want to exacerbate this distress or create additional burden or guilt by inadvertently suggesting that the cancer diagnosis is their fault because of poor lifestyle behaviours or excess body weight. Some HCPs said they would feel hypocritical raising the issue of weight management.

*HCP(F)12: I mean the patient’s already on the floor anyway. They’ve been mutilated, they’ve been radiated. They’ve lost their hair, they’ve lost their confidence, their relationships aren’t the best at the best of times… and then for somebody to chuck in, and by the way you’re fat as well… [Some of the nurses] admitted that they would feel embarrassed talking about weight gain and things like that, because they were overweight and didn’t exercise themselves. So, it was bit being a hypocrite really to give that advice.*

Some HCPs suggested strategies for broaching the issue of weight management in a sensitive way to avoid causing additional distress and feelings of guilt amongst BCPs, and focusing more effort towards the end of primary treatment.

HCP(F)1: You really have to be careful, and you have to put the conversation [about weight] in at certain points of your end of treatment conversations. You can’t just suddenly upset them with you need to lose weight. No, it’s not your endocrine, and you need to get more active. It’s very sensitively done.

HCP(F)2: [Towards the end of treatment]… their head is moving into a different place… I would say there’s definitely a shift in terms of the questions that they’re asking, and what they are open to receiving in terms of information.

Another major barrier to providing lifestyle support was the time constraints imposed on clinical appointments.

*HCP(F)9: I think to be honest it’s time constraints. I mean with the best will in the world it would be lovely to sit down with everybody for half an hour or so and chat to them, but you don’t have that. You don’t have time to do that if you’re doing everything else.*

Suggestions for overcoming the barrier imposed by time constraints, included enabling all HCPs along the care pathway to apply consistent messages via a “brief intervention” approach and/or sign-posting/referring patients onto community services, but with some reservations concerning the latter as such services can often be short-lived.

HCP(F)14: The other thing our (NHS Foundation) Trust is looking to do is to have very brief interventions in every consultation. If somebody mentions anything about smoking or alcohol or their weight or whatever, just say is that something you’d like to know more about?

HCP(F)6: It’s keeping on top of where all those services happen, because they pop up only just for short projects and then they pop back down again, so it’s knowing what sustainable services are out there that we can refer people to.

#### Support from family and friends

BCPs who reported having family and friends that provided verbal encouragement (e.g., be more active, eat healthily, etc.), were more likely to adopt healthy lifestyle behaviours during and after primary treatment. Other BCPs recounted experiences in which family and friends had discouraged them from engaging in healthy lifestyle behaviours, feeling that they had been through too much to be concerned with healthy eating and or the need for regular exercise and/or that it might be detrimental, and this negatively affected their motivation for health behaviour change.

BCP3: I think my immediate family, I think they were really supportive… my daughter… used to say yeah just get off your backside…

BCP15: Everyone around you says oh it’s OK, you’ve had cancer, have that bit of cake, and things.

#### Peer-support

Several BCPs expressed the importance of peer-support in motivating them to adopt healthy lifestyle behaviours and in their motivation for attending support sessions with similar others who they could share experiences with.

*BCP16: Everybody in the room then is feeling what you’re feeling and everybody will come up with a question and be comfortable to expand on that, because you’re all in a similar position and you can all relate to it positively, can’t you?*

For some BCPs, it was more important to move on from their cancer experience and they did not want the constant reminder of what they had been through by attending support groups or exercise classes with other patients.

*BCP1: [At the] Consett breast cancer group …they’re doing these for breast cancer ladies, these [exercise] classes, I’m a bit nervous to go, I’m like oh. And I don’t know if it’s part of the not wanting to go back into, or hang around with people with breast cancer, or whatever.*

### Theme 3: information availability for BCPs

BCPs felt poorly supported, in terms of the information they received about the potential impact of healthy lifestyle behaviours on treatment outcome and their general health and wellbeing as they progressed through the treatment pathway. This meant that if they had the motivation to adopt healthy lifestyle behaviours, they were unsure about what to do for the best and the onus was often on them to seek relevant information from other sources.

#### Patient receptiveness to information

BCPs were acutely aware of the lack of support for lifestyle behaviour change during the breast cancer treatment pathway. Some BCPs said they would have been very receptive to information (guided by HCPs) about the broader health impacts of breast cancer treatment, as well as advice on weight management via healthy eating and physical activity. The need for tailored advice and information to meet the specific needs of patients was also highlighted.

BCP8: You sort of drop off the edge of the earth really once you’ve finished your radiotherapy and you get signed off by people. That’s kind of it... then you’re on your own. Go away and exercise and lose that weight you put on, thanks.

BCP9: If I’m wanting to give my body the best fighting chance, which is what I want to do, work alongside the treatments I’ve been told I have to have, then for me it’s vital that I know what I can do to best serve my body.

L4: It’s not the same for everyone. It’s just finding the right thing for the right person, which sometimes is easier said than done.

Other HCPs said that in their experience, some patients are likely to be resistant to health behaviour change and that a tactful approach would be needed.

*HCP(F)9: The thing is that if you take a group of any population, whether they’ve got cancer or not, there would be a significant amount of people who aren’t going to move no matter what you say... maybe if we keep on and on and on at them about it it’s only going to annoy them, so you have to be very careful.*

#### The need for credible information

BCPs felt that credible information sources were essential due to the huge volume of conflicting information available on the internet that can get very confusing and may have a dubious evidence-base. In addition, many ‘myths’ or outdated information continue to circulate, such as this example from a BCP:

*BCP9: Every time you take a hit of sugar you suppress your immune system. So potentially you’re… not giving [your body] a fighting chance really. You’re suppressing that natural immunity all the time.*

Some BCPs said they were given information from HCPs in the form of leaflets but were left to interpret that information themselves and often resorted to looking up information on the internet. Some of the advice received from HCPs was perceived as vague:

*BCP15: I was told just eat a healthy diet and, yeah, not to really change anything particularly. So, it almost felt a bit vague, I felt like they weren’t really sure when I asked them the questions.*

### Theme 4: knowledge of current evidence amongst HCPs

Many HCPs said they had insufficient knowledge of the most recent scientific dietary and physical activity evidence and public health guidance to confidently discuss such matters with BCPs and would only be able to provide general advice if asked.

#### Knowledge-gap

Many HCPs discussed how they were unfamiliar with current evidence in support of the benefits of healthy eating and regular exercise for BCPs and this was clearly evident amongst BCPs.

HCP(F)9: I know that if you go for a walk it’s good for you, but I don’t know what to tell people to eat, I don’t feel I have that information. So, healthy eating just means they can go and look into it. I think you have to be a little bit careful, because if you promote the diet then it’s your responsibility to some extent. And it doesn’t work for everybody, it doesn’t, not all diets work for everybody.

BCP11: I want to try and do a healthy diet and way of living, and keep this cancer in remission. What’s the best thing that I can do? And that’s where I don’t seem to be getting a lot of information back as to what should I actually be eating?

Furthermore, BCPs lacked confidence in the ability of exercise and fitness professionals to provide breast cancer-specific advice.

*BCP3: I don’t think there’s enough, possibly not enough input of people who’ve had breast cancer into exercise classes, actually being trained to lead classes... for me it would be nice to see a programme where people who’ve had breast cancer can have the opportunity to actually be part of supporting other people coming through it, in terms of being trained up as like a....like a peer, sort of.*

The lack of evidence-based guidance underpinned a sense of nervousness and generated fears about engaging or re-engaging with exercise programmes amongst BCPs.

*BCP2: This is why, I mean this is one of the reasons why I haven’t gone back fully to exercising and doing what I want to do, because I’m so paranoid, I have a sleeve, I’m so paranoid about what it’s going to do to my arm.*

In contrast, the lymphoedema practitioners said that they use their evidence-based knowledge to empower women to be able to self-manage their lymphoedema through exercises that have been shown to be effective. They had a much better grasp of research evidence supporting the important role that exercise and weight management play in lymphoedema management. They were also more proactive in promoting this evidence to their patients, feeling it was fundamental to their role and essential for patient care. This group of HCPs were part of a specialist lymphoedema clinic (private company) commissioned to deliver the local NHS lymphoedema service for cancer and other patients. The lead nurse is an advocate of exercise and weight management and empowers her team to this end via training opportunities.

*L2: We’ve got the evidence [for the benefits of diet and exercise on lymphodema]. We can actually show them the evidence now, which a few years ago we couldn’t do that. There’s lots of evidence now for it and I think that’s our great advantage…*

#### Experiential knowledge gained

BCPs and HCPs recounted how they had gained some level of experiential knowledge about the health benefits of weight loss and regular exercise on important health outcomes following a breast cancer diagnosis. This was a source of positive motivation for some HCPs to provide lifestyle advice and for some BCPs to engage in more healthy, active lifestyles.

BCP4: I was confused for a while after treatment. I’d put on so much weight, and I was having all these joint pains and couldn’t bend over to put my shoes on without not being able to breathe... But then once I got back down below a certain weight, those things went away.

HCP(F)8: There’s people that have exercised and have clearly coped with the treatment so much better than people that haven’t I can say.

## Discussion

This qualitative study explored the experiences and perceptions of ER + BCPs and HCPs regarding weight management behaviours during and after treatment and the provision of weight management advice as part of the care pathway. During treatment, women recounted how concerns about weight management were overshadowed by the physical and emotional strain of their diagnosis and disruption to normal routines caused by hospital appointments. However, as they emerged from their primary treatment, physical changes such as increased body weight, change in body shape, shoulder mobility issues and a lack of knowledge and/or confidence to engage in healthy lifestyle behaviours (particularly physical activity and structured exercise) were important barriers to health behaviour change, consistent with previous evidence [12–15].

Studies suggest that a cancer diagnosis can act as a “teachable moment”, prompting women to adopt healthier lifestyle behaviours after a breast cancer diagnosis and/or treatment [24, 35]. However, conflicting evidence exists and the possibility of study selection bias (study samples being biased towards cancer survivors who have changed their behaviours) also needs to be taken into account [24]. Although recent evidence of modest improvements in dietary and physical activity behaviours within three years of a breast cancer diagnosis provides support for the “teachable moment” [36], improvements may not be maintained over the longer-term [37]. The low priority participants gave to weight management behaviours during treatment resonates with qualitative data from other studies [38, 39] but contrasting evidence suggests that long-standing, deep-routed concerns about excess body weight can overshadow the emotional stress of a breast cancer diagnosis in some women [40]. This demonstrates the complex interaction between the emotional consequences of a breast cancer diagnosis and existing weight management concerns. Furthermore, it emphasizes the need for weight management advice to be timely and cognizant of the balance between emotional distress and existing body weight sensitivities if the “teachable moment” is to be capitalized on by HCPs [12]. Evidence from previous studies suggests that the optimal time to implement weight management support is after primary treatment for the majority of BCPs [41, 42]. This is consistent with the views expressed by HCPs in the present study and the feeling amongst BCPs that they *“drop off the edge of the earth”* after primary adjuvant treatment and are left to their own devices to improve their lifestyles.

Our results also show that the physical and emotional barriers impeding the adoption of weight management behaviours in BCPs can be compounded by a lack of awareness, regarding the likelihood of weight gain during breast cancer treatment. Weight management in cancer patients has been dominated by concerns about unintentional weight loss secondary to treatment or progressive disease [11] and is likely to have influenced pre-treatment weigh loss expectations amongst BCPs. This could be an important barrier to the adoption of healthy lifestyle behaviours by precluding the need to seek weight management advice. In addition, women felt ill-informed and poorly supported and expressed their nervousness and fears about engaging in physical activity or structured exercise. This was due to a lack of evidence-based knowledge and guidance regarding the types of exercise it is safe to engage in, without exacerbating common side-effects, such as fatigue and lymphoedema, as reported previously [43]. There were also concerns that nutrition and exercise professionals may have insufficient experience of breast cancer and are therefore unable to provide the support that BCPs need to overcome the physical and emotional challenges experienced during recovery from primary treatment. Consistent with previous evidence [12, 43], BCPs also spoke about the conflicting information that is available through social media channels. This lack of support, in conjunction with personal sensitivities related to weight gain and other bodily changes (e.g., change in body shape, impaired shoulder mobility, etc.), underscored their lack of confidence for health behaviour change. Consequently, the need for credible, evidence-based advice and support from competent HCPs as a means of rebuilding confidence and motivation for physical activity and healthy eating, emerged as a key theme in the analysis, consistent with previous research [12, 44].

These findings highlight a training gap for HCPs, which is needed to build the required competences for the provision of evidence-based (and bespoke) weight management support to addresses the health behaviour change challenges faced by BCPs. This training and associated support programmes for BCPs should aim to develop evidence-based knowledge, while drawing on key tenets of health behaviour change theory. For example, according to the Health Belief Model, individuals are likely to adopt health behaviours when they perceive their susceptibility to an illness and its seriousness and believe that the benefits of behaviour change outweigh the perceived barriers [45]. Thus, tailored (and timely) educational support for improving knowledge of the adverse effects of weight gain and benefits to be gained from healthy dietary choices and physical activity could tip the balance in favour of behaviour change [46]. Strategies aimed at developing perceived *competence* for weight management behaviours, while being mindful of flexible options to help meet individual needs and preferences (facilitating *autonomy*), is also consistent with intrinsically motivated behaviour change according to *Self-Determination Theory* [47]. Furthermore, establishing a platform for frequent peer-to-peer contact and support would help to fulfil the need for *relatedness* (sense of belonging to a social group) in self-determined behaviour change [47]. Having the opportunity to share experiences with “similar others” is consistent with previous research, in which empathy received from women perceived to be “in the same boat” and “same as you” helped BCPs to move on from feeling isolated to feeling accepted, while also providing subtle motivational peer-pressure for health behaviour change [14, 16]. However, it is also important to note that some BCPs wanted to move on from their cancer experience and felt that this would not be served by attending weight management support groups with other patients.

The perceived role of family and friends in supporting health behaviour change was more complex, with recollections of conflicting advice and support given. While evidence suggests that support from family and friends can be a powerful motivational factor for health behaviour change in cancer patients [48], its absence and/or active discouragement from significant others (linked with “over-protection”) has been identified as a barrier to participation in physically active lifestyles in cancer patients [38, 46]. The motivation for health behaviour change amongst BCPs in the present study was influenced either in a positive or negative way, depending on the encouragement or discouragement received from family and friends during and after primary treatment. This shows that social support for health behaviour change is a complex issue for BCPs, requiring context-specific considerations. Furthermore, these findings suggest that support programmes which include family and friends in endeavours to change the health behaviours of BCPs could be beneficial [39].

Many HCPs (with the exception of the lymphoedema practitioners) said they had insufficient knowledge of up-to-date scientific dietary and physical activity evidence to confidently discuss such matters with BCPs and would only be able to provide general advice if asked. This perceived lack of evidence-based knowledge amongst HCPs is well-reported and highlights a potential training gap [49, 50]. Furthermore, because of HCP concerns about exposing patient sensitivities during weight management conversations [49, 51], interventions aimed at redressing this training gap should consider how such advice could be sensitively provided. Time constraints imposed on clinical appointments were identified as another barrier to such provision with NHS cancer care pathways, in accordance with previous findings [49], which limits delivery options to brief consultant-led conversations within the care pathway. Evidence suggests there is potential to increase the number of brief intervention consultations with appropriate training [52], consistent with the Making Every Contact Count (MECC) initiative [53], but this approach is likely to have minimal impact [54, 55]. Alternatively, having the opportunity to sign-post/refer women onto credible and motivational weight management information and support after primary treatment was viewed positively by HCPs, as in other studies [38, 42], and could help to overcome the challenge of time constraints during consultations. For these reasons, a bespoke programme of evidence-based weight management support that dovetails with the NHS breast cancer care pathway could be the optimal solution for promoting health behaviour change and developing the skills and confidence women need for effective and sustainable weight loss.

### Strengths and limitations

Participants attending the HCP focus groups came from diverse backgrounds and most professions involved in breast cancer care, including specialist nurses, oncologists and lymphoedema practitioners, enabling a broad range of relevant views. Likewise, the wide age-range of overweight BCPs involved in the focus groups (35–70 years) and varied weight management experiences allowed for a diverse representation of views. Emerging data from the BCP and HCP focus groups regarding questions and concerns around diet, physical activity and lymphoedema, dictated that further specialist input from lymphoedema practitioners was required. An additional focus group with the latter ensured that data saturation had been reached regarding all relevant topics raised by BCPs and HCPs. A potential limitation of this research was the exclusive use of focus groups as a means of understanding the perceptions of BCPs and HCPs regarding weight management support. Focus groups might be less effective for gleaning detailed information than individual interviews but capitalise on group dynamics to stimulate discussion. In the focus group sessions, new topics that may have not been explored otherwise were frequently opened or expanded upon following comments from other members of the group. However, it may have been difficult for some participants to fully express their views in the focus group setting and a mixed qualitative approach might have yielded more in-depth data.

## Conclusions

This study showed that there is a clear need for effective and timely weight management support that addresses the issues and concerns faced by women recovering from ER + breast cancer treatment. BCPs expressed difficulties accessing credible information on diet and physical activity that is relevant to the physical and psychological challenges imposed by treatment. The lack of evidence-based dietary and physical activity knowledge amongst HCPs, concerns about raising weight management issues with BCPs during treatment and time pressures on consultations present additional barriers for the provision of weight management advice. Considered together, this evidence suggests that programmes which stand apart from the NHS breast cancer care pathway and dovetail into the end of primary treatment, could provide the best route to adoptable and sustainable weight management support in this population.

## Supplementary Information

**Additional file 1.**

## Data Availability

All data generated or analysed during this study are included in this published article and its supplementary information files.

## Abbreviations

BPC: Breast cancer patient
ER +: Oestrogen-receptor positive
F: Female
HCP: Healthcare professional
L: Lymphoedema specialist
M: Male
MECC: Making Every Contact Count
NHS: National Health Service
