# Supplementary material for: The experiences and perceptions of female breast cancer patients regarding weight management during and after treatment for oestrogen-receptor positive disease: a qualitative study
Source: BMC Cancer. 2022 Nov 18;22:1189. doi: 10.1186/s12885-022-10238-7 (PMC9675095; doi:10.1186/s12885-022-10238-7)
Supplement: Supplementary file 1 — Additional file 1. [file 12885_2022_10238_MOESM1_ESM.docx]

**Supplementary table**: focus group themes and supporting quotes.

| **Theme** | **Sub-theme** | **Supporting quotes** |
| --- | --- | --- |
| **1. Treatment** | *Side-effects of treatment* | **Physiological side-effects:**  *BCP3: I think, for me I think once I got on tamoxifen I was just really hungry all the time. It literally made [me] really hungry.*  *BCP5: I’m getting really out of breath really quick just walking up the road. ...I’m absolutely wet through with sweat on top of that. So, half of the time wherever I’m going I have to take an extra set of clothes with me to get changed because I’m absolutely, my back is absolutely soaked through, literally soaked.*  *BCP14: I suffer horrendously with hot flushes and night sweats, so you’re awake probably four or five times in the night, it comes to get up in the morning you’re knackered, aren’t you?*  *BCP16: ...when they took the lymph nodes out... I had some physio[therapy]... but it didn’t work, it didn’t increase the grip in my arm... it didn’t help with the tingling and strength and everything... I do try, I try lifting, using it, but it’s only with anything that you’re not going to drop... or at work I can’t make a tray of tea for everyone because I can’t carry it with one arm.*  *BCP16: As soon as I started on the letrozole, within a week my joints were horrendous, waking me up in the night when you can’t move your legs because they’re just like set... you just get out of bed and you feel about 90, because everything hurts. So, you’ve got all this emotional stuff going on, but then you just feel shocking and as much as I’d like to exercise, I don’t particularly do much exercise.*  *BCP2: When you went to chemo and they would weigh you, and I’d be thinking oh god how many kilos have I put on this time? And so, I was partly driven by the fact that oh god I’ve put on more weight, I’ve put on more weight, but part of me just didn’t care. I could hardly taste anything... so I was just putting in my mouth anything that I could taste.*  *L2: It’s a question that we ask at assessment, whether or not they’re on tamoxifen for example. Because my belief, certainly with the years of experience of working with women, despite them saying I eat really healthily, I do exercise and yet I still can’t lose the weight. And there’s a big blame on tamoxifen.*  *HCP(F)12: There’s definitely a reduction in their metabolic rate when they’re on chemotherapy… so what they were eating previously before they can’t burn off as much and obviously, they’ve lost that muscle as well.* |
|  | *Pre-treatment expectations* | *BCP8: If only I’d known I was going to put on weight I wouldn’t have gone through the treatment.*  *BCP14: ...I think it’d be better if somebody actually said to you, somebody in authority, now look, you really need to grasp this diet and activity, because this is what’s likely to happen.*  *HCP(M)16: I think a lot of women sort of perceive that if they’re having chemotherapy that they will lose weight. The majority put on weight. And I think that’s partly because they’re less active and partly because we give them steroids, which probably some of the time. And it’s partly because they may have a few days where they don’t feel like eating anything at all, and then they over-compensate.* |
|  | *Prioritisation of treatment* | *BCP1: There’s more important things [than trying to lose weight]. You’re still here. So, this is it. It’s kind of low on the list of priorities.*  *BCP9: In your mind you’re still wanting to be this fit, healthy runner or whatever you are, you’re still that. But you’re having so much oppressed on you at that time that that just pales into insignificance.*  *HCP(F)8: I think it’s natural that they might put on weight because they’re just wiped out of energy. They’re just struggling just to come to hospital and get through the treatment, carry on with normal life. I think maybe people that might have gone to the gym before might really struggle to do it.*  *HCP(F)14: It’s very much you go to the hospital, you come home... You don’t do very much else that interferes with that. You don’t feel very well so you don’t think I’ll just nip out for five minutes and do this that and the other.* |
| **2. Support for lifestyle behaviour change** | *Support within the clinical pathway* | **Perceived lack of support amongst BCPs:**  *BCP3: I haven’t been offered anything in terms of exercise, in terms of diet, nothing, and I’m back at work making those buffets with people and eating those buffets. It would have just been nice to be offered something if you feel up to it....*  *BCP15: I think through your treatment it was more about managing the symptoms and side effects. You’ve got this problem, here’s something for it, just prescribe something else. There was never talk of try, and a friend sent me pictures of a book she’d got about a good diet, things to help constipation and that kind of thing and it was just like types of food to eat, but that didn’t come from healthcare professionals, that was never mentioned, it was just, have some tablets to help with it.*  **HCP perceived barriers to the provision of lifestyle advice/support:**  *HCP(F)1: They’re struggling through a life changing pathway of care, and the last thing they want to talk about is activity potentially and diet. It’s coming to terms to emotionally really with a breast cancer diagnosis. And this is phenomenal for them...*  *HCP(F)6: I’ve had the question, is it my lifestyle that’s cause me to have breast cancer? So, there’s that element of it as well that maybe they’re already feeling a bit guilty if you like... so again I think there’s a sensitivity around that, if we’re flagging this up at any opportunity, is there a perception that we’re blaming them for their lifestyle.*  *HCP(F)12: It’s very time constrained, so it depends on the clinic, it depends on the volume of patients of how much you can give to that patient.*  *HCP(F)13: ...Lack of time sometimes in the clinical area is a big problem. If you had more time it could be wonderful.*  *HCP(M)16: I think, and as you say our current practice with lots of patients coming through short times, relatively limited what you can do…*  **Overcoming BCP sensitivity barriers: being led by patient needs and right timing**  *HCP(F)2: I’d say 90% of the time we’re led by what they have come here for, what their issues are... And we might ask leading questions, such as are there any other issues that you’re struggling with during your treatment or… it’s about being sensitive to it. If they’re coming in to tell you how they’re dealing psychologically with something, and that’s their main focus, then.*  *HCP(F)6: ...it’s broached in terms of the side-effects and that sort of thing, and how well they’re feeling or not very well in terms of they’re able to get out and go for a little walk and fresh air. And that’s the way that it’s broached, but not directly within our assessment for treatment.*  *HCP(F)7: ...If patients bring it up, then we definitely talk to them about it.*  *HCP(F)9: The end of treatment I think is probably the right time. Because when they’re going through treatment, it’s not the right time. And if you start telling them you need to lose weight and do exercise, it gives them the message that it is their fault. And there’s so much to deal with for breast cancer patients anyway, it’s a very emotional site.*  **Overcoming the barrier imposed by time constraints: brief intervention throughout the care pathway:**  *HCP(F)1: But as well if we’re all saying the same thing, if we’re all saying at certain points through the treatment, try and eat healthily, maintain a good diet, and try and keep up with your activity. The message may get through.*  **Signposting to support services:**  *HCP(F)1: We usually say within a year of completing treatment to go on the [Moving Forward] course… The other thing I would say is that if they are receptive and you can build on that, then we have a GP referral here, they get 12 weeks free with Slimming World, and that is a huge benefit to us.*  *HCP(F)17: What I hear as well is that nurses don’t feel confident to do a lot of that referral because... services have been decommissioned... [also] some people can be asking for help but they need more than that brief intervention...*  **Other routes to support:**  *HCP(F)1: Breast cancer patients specifically have got their own breast cancer charity, and a lot of our patients go on that charity, and we use a lot of their information, and encourage them to use it as well. And if you go on the website all of this information is there.*  *HCP(F)14: I think increasingly when we’ve got our health and wellbeing events, I mean that’s where we do an awful lot of that sort of information giving [activity and diet]… but it’s only for some of the patients; it’s not for all of the patients.*  *HCP(F)1: We promote [Active Sheffield] at the end of treatment... it's a website or information leaftlet. They go onto the website, they put in their postcode, and they can identify free exercise or activities around in their vicinity where they live.* |
|  | *Support from family and friends* | **Conflicting advice and support:**  *BCP15: when I was diagnosed, I know everyone suddenly like bought me loads of chocolates and things and everyone was like oh just have that bit of cake, sod it, kind of thing. So, I knew my weight had started to creep up a little bit before I’d even had any treatment.*  *HCP(M)16: You get somebody who is told oh you must take care of yourself. No, don’t do anything. Or the other bit of come on, you must get up and be active. It’s trying to balance that, but that’s people for you.* |
|  | *Peer-support* | *BCP5: I think I’d feel better if it was kind of like a group thing. Because I think at the minute now what puts me off joining a gym or doing anything like that is my weight come on, I’m lopsided on one side because of the lumpectomy for two, and my hair for three. So, I just feel [self-conscious]*  *BCP8: If you’ve got the opportunity of a group where you might get lots of different people together, that’s helpful, with then advice around what you do. But actually where they know there are specific issues you might get...you don’t feel quite so abandoned, if that’s the right word.*  *BCP14: ...if you go to an exercise class you’ve to explain and I think I don’t want to explain, I just want to be the same as everybody that’s there."*  *BCP2: It’s really difficult isn’t it, because there are certain groups that I can’t go to now, because they just talk about cancer and I can’t bear it; whereas, there’s one group that I go to, and we talk about the abstract things around cancer, but we don’t talk about what it is like for us really, because we don’t need to... I’m in that place where I don’t want it to define me, but I want people to have an understanding of how shit it was.* |
| **3. Information availability for BCPs** | *Patient receptiveness to information* | **Evidence of BCP receptiveness:**  *BCP4: I feel like a lot of the information was targeted at an older audience, like older ladies. And there’s so many younger women now being diagnosed, so I feel like that’s where I wasn’t supported, because I was given information that wasn’t relevant to me.*  *BCP10: They gave you a leaflet... nobody talked you through it, they just gave you the leaflet and said read that, do these exercises. And I did say to them but I do walking, and they said well as soon as you feel fit to go back walking again go back. But that was all really I did have.*  *BCP11: I want to try and do a healthy diet and way of living, and keep this cancer in remission. What’s the best thing that I can do? And that’s where I don’t seem to be getting a lot of information back as to what should I actually be eating?*  *HCP(F)2: [At six months or end of treatment point] their head is moving into a different place... So I would say there’s definitely a shift in terms of the questions that they’re asking, and what they are open to receiving in terms of information.*  *HCP(F)14: I do find too there’s increasing numbers of younger women actually asking at the time of diagnosis what can I do now? In the next few weeks, what can I do to make things better for myself?*  *HCP(M)16: A not uncommon question from patients towards the end of their treatment is what can I do? And in one respect, I mean that’s obviously an opportunity.*  **Need for tailoring the information:**  *BCP11: When I asked the questions I got answers from the breast care nurse, I got answers from the oncologist. But actually, if I’d have probably seen a dietician, nutritionist, and you can talk about your specific cancer, because really that’s all we’re interested in, isn’t it? How does that affect me?*  *BCP15: I feel like I’ve got no core strength or anything because I’ve been doing nothing, some kind of like Pilates or yoga or something, but for people maybe who’ve had cancer, because I can’t really find anything that isn’t just a general public session and, like I said, I don’t want to have that conversation with an instructor.*  **Not for all patients:**  *HCP(F)1: Patients have got to want to engage with it haven’t they? ...I think it’s about actively listening to your patient isn’t it, and hearing what they want. Yes, we’ve got to give them all of this healthy advice about getting back to normal, but it’s about what people want to hear.*  *L2: You’ve got two camps usually, you’ve got some will say oh yeah I’m happy to do anything, I really want to do it, and there are others who are no chance, no way am I getting in that swimming pool, forget it.* |
|  | *The need for credible information* | *HCP(F)7: Quite a lot are asking about what kind of diets. And they’ve read oh maybe I shouldn’t eat this and not eat that. So, I think there’s more internet searching going on by patients thinking about what they need to not eat. So, we get asked about that quite a lot I’d say during treatment.*  *BCP11: There’s a lot of conflicting information out there, whether that be on internet or reading it or just talking to people. People have said to me oh you need to go vegan. But then I’m thinking well that’s fine, but what about the dairy for your bones and everything like that. So, I’ve got conflicting information about that.* |
| **4. Knowledge of current evidence amongst HCPs** | *Knowledge-gap* | **Limited knowledge of the evidence-base amongst HCPs:**  *BCP9: I asked the oncologist what about phytoestrogens? And they went no, no, no, stay away from anything with oestrogen in. I then said can I have soya milk then? And he went yes of course you can have soya milk, you don’t have to worry about your soya milks. I went soya milk has got phytoestrogens in it. He went oh, no stay away from everything with oestrogens in.*  *BCP9: I mean I would take all the medication that I was given, which I’m doing, but as well as that I want to help myself as much as I can via diet, via exercise, and that’s where I don’t seem to be getting an awful lot of feedback to me. Just tell me what I should really be doing.*  *BCP11: ...I want to try and do a healthy diet and way of living and keep this cancer in remission. What’s the best thing that I can do? And that’s where I don’t seem to be getting a lot of information back as to what should I actually be eating?*  *HCP(M)10: A lot of patients maybe stopped from exercising or dieting because of the direct side effects from the medication... joint aches and pains, which is quite common in breast cancer tablets. And I don’t know if there’s any evidence that exercise actually might improve those symptoms, is there?*  *L2: I think a lot of [patients] don’t know how to eat healthily. They haven’t got that knowledge... I was absolutely astounded that people didn’t know how to eat healthily, that they had to have fruit and vegetables as part of their daily intake. They didn’t know how to make a basic soup.*  **Lack of confidence in exercise professionals:**  *BCP9: [It would be helpful to learn from women who have] suffered breast cancer themselves, and they’re personal trainers. I follow a lady who has just had the removal of implants, and she’s a personal trainer. And so she walks you through her journey about what exercise can I do now...*  *BCP12: I feel like if there was any group like for health and wellbeing after cancer treatment... and where you can do maybe exercises or people who know about cancer patients and... you don’t have to explain so much, that would be helpful...*  *BCP3: I went to a boot camp with my daughter. And it was an ex-army guy doing the boot camp... I couldn’t do some of the stuff, because I really was out of breath... and because I couldn’t do it, if someone couldn’t do it he made everybody do it again. And that was humiliating.*  **Nervousness and fears about engaging in exercise:**  *BCP12: …part of me feels restless and I want to get back to how I used to be and start getting on with my life, work, you know usual, however I managed, but I’m so nervous. I don’t want to go to a class, like before I could do Zumba and it was fun and now I don’t know that I might be more ill after, so I don’t know what is OK for me after all these things that has happened.*  *L3 ...they tell you not to do things... But I think you can be too cautious around that. For me it’s about feeling good, feeling fit, because really I could have just stayed in the house on tamoxifen for five years and not done anything. And I don’t think that’s, that’s no life is it? You want to have a life.*  *BCP11: I want to know what can I do, how much can I do? And everybody keeps saying to me just do what you think. Well actually I’d like to increase it, but he’s saying to me no, you’re going to have to do that extremely steadily, I don’t want you increasing even by five minutes... I said is consistency better than quantity? And he went just do it when you want... standing a bit back [I'm now] thinking I don’t really know what I’m doing...*  *BCP15: I’m not back at the gym yet because I don’t quite know what I can do, like I used to do weights and stuff like that and I’m a bit nervous at the moment of what I can manage. I used to go to classes and I’m worried going into a class and then trying it and going oh I can’t do it, and having to walk out or something.*  **Lymphoedema support as an exception:**  *L3: The whole point is that we end up with expert patients that can be safely discharged from the clinic and that can manage their own care...*  *L2: I think because we’re quite knowledgeable in what we do and we keep up to date with as much information as possible, we do know that weight is a big part of their pathway, of their journey.*  *L2: What we do when we see them is to start from scratch and to build up their knowledge about lymphoedema so that they know exactly what to do for themselves.* |
|  | *Experiential knowledge gained* | *BCP2: I know that there is a certain weight which is about 10 pounds lighter than I am at the moment that my backache goes. And I know that this is backache, this is overweight backache, not tamoxifen backache.*  *BCP4: I hadn’t realised how much alcohol I was drinking. So I was, I’d got to the point where I was having a glass of wine every day, or like a little bottle of lager, and for some reason I just thought I just need to stop. So I cut it out, and within four weeks I’d lost about 4kg just from cutting out alcohol.*  *BCP8: I’m very careful about what I eat. So, we don’t have fizzy drinks in the house. We don’t have chocolate biscuits in the house. We’ve got crisps, but I don’t eat them. And I only have a couple of glasses of wine at a weekend – spent a lot of my life not drinking at all.*  *HCP(F)12: There’s definitely a reduction in their metabolic rate when they’re on chemotherapy… so what they were eating previously before they can’t burn off as much and obviously they’ve lost that muscle as well.*  *HCP(F)14: You lose muscle mass and you gain adipose tissue, and it’s where you gain the adipose tissue that makes you feel and look as if you’ve gained weight, and actually perhaps your weight’s actually remained the same.*  *L1: And so it’s finding out what their habits are, what their triggers are, and then very gently encouraging them to get into just healthy eating habits... so, it’s having breakfast, it’s eating sensible meals, increasing fruit, increasing fibre, increasing vegetables, cutting down on the fat, cutting down on the sweets. It’s very general things.* |
